# Job satisfaction, burnout and absenteeism-illness of workers in a public university hospital

**DOI:** 10.47626/1679-4435-2026-1474

**Published:** 2026-02-27

**Authors:** Carla Patricia Antunes Gontijo, Silmar Maria da Silva, Janaina Soares, Michele Nunes Mesquita, Roany Cistellis Silva Domingos, Maria Odete Pereira

**Affiliations:** 1 Universidade Federal de Minas Gerais, Escola de Enfermagem, Belo Horizonte, MG, Brazil; 2 Universidade Federal de Minas Gerais, Escola de Enfermagem, Departamento de Enfermagem Aplicada, Departamento de Enfermagem Básica, Belo Horizonte, MG, Brazil

**Keywords:** psychological exhaustion, job satisfaction, occupational health, teaching hospitals, absenteeism., esgotamento psicológico, satisfação no emprego, saúde ocupacional, hospitais de ensino, absenteísmo.

## Abstract

**Introduction:**

Sickness absenteeism impacts the healthcare workforce and may be related to job satisfaction and burnout.

**Objectives:**

To analyze the association between job satisfaction and burnout syndrome in a public university hospital.

**Methods:**

Cross-sectional study, carried out between January and February 2022. A sociodemographic questionnaire, the Job Satisfaction S20/23, and the Burnout Characterization Scale were used for data collection. Secondary data were obtained through medical certificates. The descriptive and multivariate analysis of statistical data considered a significance level of 5%.

**Results:**

There was a statistically significant correlation between sickness absenteeism and job satisfaction, since the lower the satisfaction in hierarchical relationships (p = 0.001) and the lower the intrinsic job satisfaction (p = 0.000), the higher the sickness absenteeism, and the higher the job satisfaction, the lower the burnout (p < 0.05).

**Conclusions:**

It can be inferred that the greater the job satisfaction, the lower the burnout and the lower the absenteeism-illness.

## Abstract (pt)

**Introdução:**

O absenteísmo-doença impacta a força de trabalho na saúde, podendo estar relacionado à satisfação profissional e ao *burnout*.

**Objetivos:**

Analisar a associação entre a satisfação profissional e a síndrome de *burnout* de um hospital universitário público.

**Métodos:**

Tratou-se de um estudo transversal, realizado entre janeiro e fevereiro de 2022. Para a coleta de dados, foram utilizados um questionário sociodemográfico, a Satisfação no Trabalho S20/23 e a Escala de caracterização do *burnout*. Os dados secundários foram obtidos por meio de atestados. A análise descritiva e multivariada dos dados estatísticos considerou a significância de 5%.

**Resultados:**

Verificou-se correlação estatisticamente significativa entre absenteísmo-doença e satisfação no trabalho, visto que, quanto menor a satisfação em relações hierárquicas (p = 0,001) e menor a satisfação intrínseca no trabalho (p = 0,000), maior o absenteísmo-doença e que, quanto maior a satisfação no trabalho, menor o *burnout* (p < 0,05).

**Conclusões:**

Pode-se inferir que quanto maior a satisfação no trabalho, menor o *burnout* e menor o absenteísmo-doença.

## INTRODUCTION

Professional satisfaction is a pleasant emotional state that arises from concluding that one’s job facilitates the achievement of human values. Individuals perceive satisfaction based on what they consider important and the way they perceive the results ^[1]^. Job satisfaction can positively influence the workforce’s efforts ^[2]^. The work environment influences professional satisfaction and resonates in other dimensions of organizational behavior, such as organizational commitment, professional performance, intention to quit the job or the profession, employee turnover, and stress and burnout ^[3]^.

Health care professionals, especially those who work in hospitals, experience stressful situations on a daily basis, including pain, suffering, and death. They are also exposed to an intense work pace, shift work, long work shifts, low salaries, complicated interpersonal relationships, a lack of material resources, and inadequate staffing, among other factors that can exacerbate work-related stress ^[4]^.

Burnout syndrome results from chronic, unsuccessfully managed workplace stress ^[5]^. It is characterized by emotional exhaustion, dehumanization and disappointment with work ^[6]^, as well as dissatisfaction with hierarchical relations, dissatisfaction with the work environment, and low intrinsic job satisfaction ^[7]^.

Among health care workers, risk factors for burnout include: 1) environmental factors in the hospital context (occupational risk exposure, work process characteristics, and quantitative and qualitative work overload); 2) environmental factors in intensive care units (complex locations and care for critically ill patients who require constant monitoring and have a high mortality risk); 3) social factors in the hospital context (interpersonal conflicts); and 4) personal factors (personality, self-esteem, and coping resources) ^[8]^.

Due to its consequences for health (such as depression) and socioeconomics (turnover, increased social security costs, and absenteeism), burnout syndrome should be considered a public health problem ^[9]^. Sickness absenteeism refers to a worker’s absence due to illness or medical procedures. Its multifactorial etiology originates in individual, interpersonal, environmental, and/or organizational factors, and it may be related to the position or function, the work environment, and biological, psychological, and psychosocial factors ^[10]^.

Aiming at increased professional satisfaction and decreased burnout, and given the pandemic’s reshaping of health care work, health managers needed to know the quantitative and qualitative dimension of sickness absenteeism and identify its associated factors (interpersonal and personal factors and work conditions and processes) before they could plan effective interventions ^[9]^. Thus, the question arose: what is the relationship between professional satisfaction, burnout syndrome, and sickness absenteeism among public university hospital workers?

Understanding this relationship could help broaden reflections on the labor activity of health professionals who work in hospitals and help guide initiatives to improve satisfaction and reduce worker illness. The present study investigated the association between professional satisfaction and burnout syndrome at a public university hospital.

## METHODS

### STUDY DESIGN, PERIOD, AND LOCATION

This cross-sectional study investigated the relationships between professional satisfaction, burnout syndrome, and sickness absenteeism, based on Strengthening the Reporting of Observational Studies in Epidemiology guidelines for cross-sectional studies ^[11]^. Data were collected in January and February 2022 at a public university hospital, the Federal University of Minas Gerais (UFMG) Hospital das Clínicas (HC-UFMG) a reference center for mediumand high-complexity care in Belo Horizonte, capital of Minas Gerais, Brazil.

### POPULATION AND ELIGIBILITY CRITERIA

The study population consisted of 1,667 public employees of the Brazilian Hospital Services Company (EBSERH) and 1,111 statutory civil servants of UFMG, totaling 2,778 permanent workers, according to data provided by the HC-UFMG Personnel Management Division in December 2021.

A stratified random sampling strategy was used. The target population units were identified, with similar units gathered into subgroups or strata. ^[10]^ Employees were drawn from each professional category: physicians, nurses, nursing assistants and technicians, higher-level care positions (such as physical therapists and psychologists), technical-level care positions (such as radiology and pharmacy technicians), higher-level administrative positions, secondary and technical-level administrative positions, leadership, and support workers (such as pantry workers and telephone operators). Using OpenEpi 3.0.1, the sample size was calculated at 338 people.

Permanent staff during the data collection period were included in the study, ie, those who had an employment relationship with EBSERH or were statutory civil servants of UFMG, regardless of the length of service, workplace, or position, provided they had been hired by December 2020 or were on health leave < 6 months, given the representativeness of sickness absenteeism in this study. Workers on leave without pay, on maternity leave, or on leaves > 6 months during the data collection period were excluded from the sample. After applying these criteria, the number of eligible workers was 925.

### DATA COLLECTION PROCEDURES

The workers selected to participate in the study were invited via text message with a link to the informed consent form and the questionnaires, either through a hidden list or individually. Collection was suspended after reaching the necessary number of participants in each stratum. The responses of all participants who met the inclusion criteria were considered, even surplus cases within each stratum.

Three electronic instruments were used for primary data collection: 1) a semi-structured questionnaire that sociodemographically, economically, and professionally characterized the involved workers; 2) the Job Satisfaction Questionnaire S20/23, a reduced version of the Job Satisfaction Questionnaire S4/82, which has been adapted and validated for Brazil ^[7]^ and consists of 20 items in 3 dimensions (satisfaction with hierarchical relations, satisfaction with the work environment, and intrinsic job satisfaction); and 3) the Burnout Characterization Scale, which was designed and validated in Brazil ^[6]^ and consists of 35 items in 3 dimensions (emotional exhaustion, dehumanization, and disappointment with work).

Secondary data on sickness absenteeism were collected in spreadsheets, based on medical and dental certificates presented between January 2016 and December 2020. This period was chosen due to the availability of absenteeism data at the host institution. The percentage of sickness absenteeism was measured for each worker (number of days permitted on the leave certificate/s in relation to the number of scheduled work days). The number of scheduled work days was calculated according to the number of days in each calendar year, with respect to the worker’s hire date.

### DATA ANALYSIS

The data were analyzed through descriptive analysis, with frequency and proportion for categorical variables. The Shapiro-Wilk test was used to determine data normality for quantitative variables ^[12]^, which are presented as median and quartiles. The median sickness absenteeism from 2016 to 2020 was calculated to treat absenteeism as a single variable and analyze the association between satisfaction variables and work absences.

The Mann-Whitney test was used to compare test results between groups. The Kruskal-Wallis test was performed for comparisons involving ≥ 3 groups. Spearman’s correlation was used when the 2 quantitative variables were analyzed. Finally, the Friedman test was used to for longitudinal assessment of absenteeism ^[12]^. All analyses were performed in IBM SPSS Statistics 25, with a significance level of 5%.

### ETHICAL ASPECTS

This study was approved by the UFMG research ethics committee (opinion 5.104.069) and the HC-UFMG research ethics committee. Participant consent was obtained through an informed consent form, guaranteeing their participation in the research in an autonomous, conscious, free, and informed manner. A representative of the participating institution signed a data use commitment form, allowing access and use of the sickness absenteeism data in spreadsheets.

## RESULTS

The sample consisted of 452 workers, of whom 114 were nursing assistants and technicians, 100 were nurses, 101 were physicians, 49 were administrators, 39 were higher-level care professionals, 21 were secondary-level care workers, 19 were in leadership, and 9 were support workers. Most were women (74%), lived with a partner (66.2%), had a mean age of 43 years, were employed through EBSERH (68.4%), worked only at HC-UFMG (68.6%), had worked at the institution between 5 and 10 years (50.7%), worked on the day shift (76.7%), worked 30 hours per week (64.4%) and ≤ 8 hours per day (62.4%).

The median scores for each Job Satisfaction Questionnaire domain were: satisfaction with hierarchical relations - 3.09 points, satisfaction with the work environment - 3.20 points, and intrinsic job satisfaction - 3.50 points. Satisfaction with hierarchical relations had the lowest score among participants and intrinsic job satisfaction had the highest score (Table 1).

**Table 1:** Quartile distribution for the Job Satisfaction Questionnaire S20/23 dimensions

JSQ domains	n	Q1	Median	Q3
IJS	452	2.56	3.50	4.00
SWE	452	2.40	3.20	4.00
SHR	452	2.30	3.09	3.82

JSQ = Job Satisfaction Questionnaire; SWE = satisfaction with the work environment; IJS = intrinsic job satisfaction; SHR = satisfaction with hierarchical relations.

The median scores for each Burnout Characterization Scale dimension were: dehumanization - 1.50 points, disappointment with work - 2.00 points, and emotional exhaustion - 2.83 points (Table 2).

**Table 2:** Quartile distribution for the dimensions of the Burnout Characterization Scale

BCS domains	n	Q1	Median	Q3
Emotional exhaustion	452	2.25	2.83	3.58
Dehumanization	452	1.20	1.50	1.90
Disappointment with work	452	1.54	2.00	2.62

BCS = Burnout Characterization Scale

Our analysis was based on sick leave certificates presented between 2016 and 2020, due to data availability for the institution’s occupational health teams. The sickness absenteeism index was calculated for each worker by measuring the number of days lost due to medical and dental problems in relation to the number of scheduled work days that year (Table 3).

**Table 3:** Longitudinal assessment of sickness absenteeism

2016	2017	2018	2019	2020	p-value
0.27 (0-2.19)	0.27 (0-1.92)	0.55 (0-2.74)	0.55 (0-3.01)	1.78 (0-4.92)	0.0000

* Friedman test for paired groups. There was a significant difference between 2020 and the other years.

The median percentage of lost work days in 2016 and 2017 was 0.27%, which is approximately 1 day of leave per year per worker. In 2018 and 2019, the median value rose to 0.55% (2 days of leave per year per worker). In 2020, the median value rose again to 1.78% (6.5 days of leave per worker).

There was a significant negative correlation (p < 0.05) between all burnout scale dimensions and satisfaction scale dimensions; hence, the higher the satisfaction, the lower the burnout (Table 4).

**Table 4:** Correlation between burnout, job satisfaction, and sickness absenteeism

BCS Dimensions		IJS	SWE	SHR	Absenteeism
Emotional exhaustion	Correlation coefficient	-0.567^^**^^	-0.305^^**^^	-.493^^**^^	0.213^^**^^
	p-value	0.000	0.000	0.000	0.000
Dehumanization	Correlation coefficient	-0.247^^**^^	-0.150^^**^^	-0.292^^**^^	-0.106^^**^^
	p-value	0.000	0.001	0.000	0.024
Disappointment with work	Correlation coefficient	-0.664^^**^^	-0.248^^**^^	-0.528^^**^^	0.109^*^
	p-value	0.000	0.000	0.000	0.021

BCS = Burnout Characterization Scale; SWE = satisfaction with the work environment; IJS = intrinsic job satisfaction; SHR = satisfaction with hierarchical relations.

* Correlation significant at the 5% level.

** Correlation significant at the 1% level.

Absenteeism was positively correlated with exhaustion and disappointment with work, ie, the greater the exhaustion and disappointment, the greater the absenteeism. However, dehumanization had a negative coefficient; hence, the greater the dehumanization, the lower the absenteeism.

## DISCUSSION

Upon evaluating the participants’ job satisfaction, satisfaction with hierarchical relations was the most impaired dimension, which can affect worker health, care quality, and even the intention to remain in the institution and the profession. Similar results regarding job satisfaction were found in a study on nursing professionals at a university hospital, in which the median satisfaction with hierarchical relations was 3.27, the median satisfaction with the work environment was 3.20, and the median intrinsic job satisfaction was 3.50 ^[13]^.

Comprehension of the factors involved in job satisfaction contributes to better decision-making since hierarchical relations in hospitals usually reflect a techno-bureaucratic organizational structure that tends toward rigid and autocratic relationships ^[14]^. A complex and hostile work environment not only affects satisfaction level of workers, but also their personal and professional needs. This has repercussions for their physical and mental health, causing stress at work.

Extreme situations of chronic and continuous stress trigger burnout syndrome ^[15]^. A study identified the following mean results in burnout dimensions: dehumanization - 1.51, disappointment with work - 2.12, and emotional exhaustion - 1.65 ^[16]^. The high scores in the emotional exhaustion dimension are probably due to greater ease in sharing feelings of weariness and stress than admitting attitudes of indifference toward patients or accepting feelings of low professional and personal accomplishment ^[6]^. Professionals with a tendency toward burnout syndrome reported feeling less satisfied with work and requested more medical leaves than those without this tendency ^[17]^.

There was a significant difference in absenteeism before and during the COVID-19 pandemic (ie, 2020). The increase in missed work days during the pandemic was directly related to COVID-19 symptoms, both suspected and confirmed cases. The higher absence rate may also have been caused by stress and fatigue due to work overload ^[18]^. A study on with workers at a university hospital found a sickness absenteeism rate of approximately 3.25% between 2014 and 2020, being 2.97% in the pre-pandemic period and 5.10% during the pandemic. During the pandemic, average sickness absenteeism days per month were 2.03 times higher (1,259 days) than in the pre-pandemic period (619 days). Among the 1,035 staff members working in December 2020, an average of 14.6 days was lost per person that year ^[18]^.

The responsibility for absenteeism does not always lie with the worker, since deficient supervision, repetitive tasks, demotivation, and discouragement, unfavorable environmental and working conditions are organizational factors, as is the precarious integration between employees and the organization, in addition to the psychological effects of management that does not promote educational and humanistic policies ^[19]^. When there is a good relationship between workers and supervisors, shift exchanges and time bank compensations are often authorized and facilitated, potentially preventing workers from seeking medical care and taking sick leaves. However, in health institutions with inflexible policies, these strategies are not viable, resulting in higher rates of absenteeism and sick leaves.

Further studies are needed on work-related factors at HC-UFMG that result in higher sickness absenteeism among full-time workers. The requirement for different types of knowledge and skills with various degrees of demand could lead to greater absenteeism among workers who are not exclusively employed by HC-UFMG, potentially increasing overload and resulting in sick leaves.

Work recognition by management, coworkers, patients, and the workers themselves has repercussions on worker perceptions; thus, it is inferred that job satisfaction is related to the prevalence of burnout, given that professionals who do not identify with the workplace are also affected ^[15]^. There is a negative correlation between job satisfaction and burnout syndrome, ie, the more dissatisfied, the greater the burnout ^[7]^.

A study on nurse managers and assistants in primary care found that, regardless of job position, intrinsic work satisfaction is strongly related to all dimensions of exhaustion. Inherent job satisfaction is likely declining due to a lack of career growth opportunities, resulting in dissatisfied professionals ^[20]^.

A study on emergency department workers in 6 hospitals in Medellin and Bogotá, Colombia found a negative correlation between burnout syndrome and job satisfaction, showing that stress disproportionately and inversely affects job satisfaction; ie, the higher the stress level, the lower the motivation ^[15]^.

In a study of 1,257 Chinese health professionals from 34 hospitals equipped with clinics or wards for patients with COVID-19, a considerable proportion of the participants reported symptoms of depression, anxiety, insomnia, and distress. Nurses, women, frontline health professionals (ie, those involved in direct diagnosis, treatment, and care of patients with COVID-19), and those working in Wuhan, China, reported more severe degrees of all mental health impairment indicators than other health professionals ^[21]^.

Chronic stress, exhaustion, and worker burnout from intense workloads worsened during labor shortages caused by COVID-19-related absenteeism, intensifying the workers’ feelings of powerlessness when facing the severity of cases and a lack of beds and/or life support equipment ^[22]^.

The COVID-19 pandemic increased tension between management and health care workers. Extra shifts and unsafe working conditions were added to previous problems, such as initiatives to improve worker efficiency and care quality. Therefore, to align physicians and administrators during the pandemic, it was recommended that leadership maintain transparency, disclosing information and updates regarding case analysis and trends, management, clinical guidelines, and supply status. Access to such information would result in greater trust, showing that leaders were making decisions based on the best scientific evidence and sources, rather than prioritizing financial interests during this crisis ^[23]^.

Regular worker meetings were held to cultivate relational connections and help alleviate care-related burdens. The meetings were a space for self-reflection, mindfulness, and self-care. Considered a valid strategy for improving working conditions and interpersonal relationships, these meetings also added a sense of value and teamwork ^[24]^.

## CONCLUSIONS

When analyzing the association between professional satisfaction and burnout syndrome at a public university hospital, a significant negative correlation (p < 0.05) was found between all dimensions of the burnout scale and the satisfaction scale; hence, the higher the job satisfaction, the lower the burnout. Absenteeism was positively correlated with exhaustion and disappointment with work; hence, the greater the exhaustion and disappointment, the greater the absenteeism. However, dehumanization had a negative coefficient; hence, the greater the dehumanization, the lower the absenteeism. Thus, it can be inferred that the higher the job satisfaction, the lower the burnout and the lower the sickness absenteeism.

## Data Availability

Upon publication, the data will be made available by the authors upon request.
